# Analysis of IgG4 class switch-related molecules in IgG4-related disease

**DOI:** 10.1186/ar3924

**Published:** 2012-07-23

**Authors:** Hiroto Tsuboi, Naomi Matsuo, Mana Iizuka, Sayaka Tsuzuki, Yuya Kondo, Akihiko Tanaka, Masafumi Moriyama, Isao Matsumoto, Seiji Nakamura, Takayuki Sumida

**Affiliations:** 1 Department of Internal Medicine, Faculty of Medicine, University of Tsukuba, 1-1-1 Tennodai, Tsukuba, 305-8575, Japan; 2 Faculty of Dental Science, Kyushu University, 3-1-1 Maidashi, Fukuoka, 812-8582, Japan

## Abstract

**Introduction:**

Immunoglobulin G4 (IgG4)-related disease (IgG4-RD) is a new disease entity characterized by high serum IgG4 levels, IgG4-positive plasmacytic infiltration, and fibrosis in various organs. The purpose of this study was to determine the mechanism of upregulation of IgG4 class switch recombination in IgG4-RD.

**Methods:**

We extracted RNA from peripheral blood mononuclear cells (PBMCs) of patients with IgG4-RD (*n *= 6), Sjögren syndrome (SS) (*n *= 6), and healthy controls (*n *= 8), from CD3-positive T cells and CD20-positive B cells sorted from PBMCs of patients with IgG4-RD (*n *= 3), SS (*n *= 4), and healthy controls (*n *= 4), as well as from labial salivary glands (LSGs) of patients with IgG4-RD (*n *= 11), SS (*n *= 13), and healthy controls (*n *= 3). The mRNA expression levels of IgG4-specific class switch-related molecules, such as Th2 cytokines (IL-4 and IL-13), Treg cytokines (IL-10 and TGF-β), and transcriptional factors (GATA3 and Foxp3) were examined with quantitative polymerase chain reaction (PCR). IgG4-nonspecific class switch-related molecules, such as CD40, CD154, BAFF, APRIL, IRF4, and AID, were also examined.

**Results:**

The expression levels of Treg cytokines (IL-10 and TGF-β) and AID were significantly higher in LSGs of IgG4-RD than in SS and the controls (*P *< 0.05, each). In contrast, those of CD40 and CD154 were significantly lower in PBMCs of IgG4-RD than in SS (*P *< 0.05, each), whereas CD40 in CD20-positive B cells and CD154 in CD3-positive T cells were comparable in the three groups.

**Conclusion:**

Overexpression of IL-10, TGF-β, and AID in LSGs might play important roles in the pathogenesis of IgG4-RD, such as IgG4-specific class-switch recombination and fibrosis. IgG4 class-switch recombination seems to be mainly upregulated in affected organs.

## Introduction

IgG4-related disease (IgG4-RD) is a new disease entity characterized by high serum IgG4 levels, infiltration of IgG4-positive plasmacytes, and fibrosis of various organs, such as pancreas, bile duct, salivary and lacrimal glands, thyroid, lung, liver, kidney, prostate, aorta, retroperitoneum, and lymph nodes [1,2]. Although the clinical features including serum abnormalities, organ involvement, diagnosis, and the therapeutic approach have been reported recently [1,2], the pathogenesis of this disease, including the roles of high IgG4 and IgG4-positive plasmacytes, and the molecular mechanism involved in the upregulation of IgG4 class-switch recombination remains unclear.

Recent studies reported increased proportions of type 2 helper T (Th2) cells and regulatory T (Treg) cells and increased production levels of Th2 and Treg cytokines in tissues of IgG4-RD, such as sclerosing pancreatitis and cholangitis [3], sialadenitis [4,5], and tubulointerstitial nephritis [6]. Moreover, high Th2 cell count and overproduction of Th2 cytokines have been described in peripheral blood of IgG4-RD [4,7], as well as in Treg cells [8].

It is reported that Th2 cytokines (IL-4 and IL-13) and Treg cytokine (IL-10) can induce IgG4- and IgE-specific class-switch recombination [9,10], and tumor growth factor (TGF)-β, a Treg cytokine, could induce tissue fibrosis [11]. Thus, increased production of IL-4, IL-13, IL-10, and TGF-β could contribute to the pathogenesis of IgG4-RD, including high serum IgG4 level, IgG4-positive plasmacytic infiltration, and fibrosis.

Conversely, CD40, CD154, B-cell activating factor belonging to the tumor necrosis factor family (BAFF), a proliferation-inducing ligand (APRIL), interferon regulatory factor 4 (IRF4), activation-induced cytidine deaminase (AID), and IL-21 contribute to nonspecific immunoglobulin class-switch recombination (from IgM to IgG1, IgG2, IgG3, IgG4, IgA, and IgE) [12-14], as well as IL-4, IL-13, IL-10, and TGF-β. The roles of these nonspecific immunoglobulin class switch-related molecules in the pathogenesis of IgG4-RD have not been reported previously.

To determine the mechanism of upregulation of IgG4 class-switch recombination in IgG4-RD, the mRNA expression levels of IgG4-specific and nonspecific class switch-related molecules were first measured in peripheral blood mononuclear cells (PBMCs), CD3-positive T cells, and CD20-positive B cells sorted from PBMCs, as well as labial salivary glands (LSGs) of IgG4-RD. Then, these levels were compared with those measured in patients with Sjögren syndrome (SS) and in healthy controls.

## Materials and methods

### Study population

PBMC samples were collected from six Japanese patients with IgG4-RD, as well as from six Japanese patients with SS who had been followed up at the University of Tsukuba Hospital (Ibaraki, Japan). LSG samples were also collected from 11 Japanese patients with IgG4-RD and 13 Japanese patients with SS who had been followed up at the University of Tsukuba Hospital and Kyushu University Hospital (Fukuoka, Japan). All patients with IgG4-RD satisfied the comprehensive diagnostic criteria for IgG4-related disease (IgG4-RD) 2011 proposed by the All Japan IgG4 team [15]. The diagnosis of IgG4-RD was based on the presence of all three items: (1) clinical examination showing characteristic diffuse/localized swelling or masses in single or multiple organs, (2) hematologic examination showing elevated serum IgG4 concentrations (135 mg/dl), and (3) histopathologic examination showing (a) marked lymphocyte and plasmacyte infiltration and fibrosis; (b) infiltration of IgG4^+ ^plasma cells: ratio of IgG4^+^/IgG^+ ^cells >40% and >10 IgG4^+ ^plasma cells/HPF. All patients with SS satisfied the Japanese Ministry of Health criteria for the diagnosis of SS (1999) [16]. These criteria included four clinicopathologic findings: lymphocytic infiltration of the salivary or lacrimal glands, dysfunction of salivary secretion, keratoconjunctivitis sicca, and presence of anti-SS-A or -SS-B antibodies. The diagnosis of SS was based on the presence of two or more of these four items. We also collected control samples: PBMCs from eight and LSGs from three healthy subjects. Approval for this study was obtained from the local ethics committee, and a signed informed consent was obtained from each subject.

### Sorting of CD3-positive T cells and CD20-positive B cells from PBMCs with flow cytometry

PBMCs derived from IgG4-RD (*n *= 3), SS (*n *= 4), and controls (*n *= 4) were stained with anti-CD3 antibody conjugated with FITC (BioLegend, San Diego, CA, USA) and anti-CD20 antibody conjugated with APC (BioLegend). Stained PBMCs were analyzed and sorted with flow cytometry.

### Analysis of mRNA expression levels of IgG4-specific and nonspecific class switch-related molecules in PBMCs and LSGs

Total RNA was extracted from PBMCs, CD3-positive T cells, and CD20-positive B cells sorted from PBMCs, as well as LSGs, by the ISOGEN method, and cDNA was synthesized by a cDNA synthesis kit (Takara, Otsu, Shiga, Japan). The mRNA expression levels of IgG4-specific class switch-related molecules, such as Th2 cytokines (IL-4 and IL-13), Treg cytokines (IL-10 and TGF-β), and transcriptional factors (GATA3 and Foxp3) were examined with quantitative PCR. IgG4-nonspecific class switch-related molecules, such as CD40, CD154, BAFF, APRIL, IRF4, and AID also were examined. The human glyceralaldehyde-3-phosphate dehydrogenase (GAPDH) was examined as an internal control.

### Statistical analysis

Differences between groups were examined for statistical significance by using the Mann-Whitney *U *test, whereas differences in frequencies were analyzed with the Fisher Exact probability test. A *P *value less than 0.05 denoted the presence of a statistically significant difference.

## Results

### Clinical and pathologic features of patients and controls

Table 1 summarizes the clinical and pathologic features of participating subjects. The frequencies of anti SS-A antibodies and anti SS-B antibodies were significantly lower in IgG4-RD than in SS (*P *< 0.05; Fisher Exact probability test). CH50 levels were also significantly lower in IgG4-RD than in SS (*P *< 0.05, Mann-Whitney *U *test). Table 2 shows material sampling and organ involvements in patients with IgG4-RD. All LSG samples used in this study satisfied histopathologic findings of the comprehensive diagnostic criteria for IgG4-related disease (IgG4-RD) proposed in 2011 by the All Japan IgG4 team [15]. As shown in Table 2, seven of 15 IgG4-RD patients showed salivary and lacrimal glands involvement alone, whereas the others had multiple organ involvements.

**Table 1:** Clinical and pathologic features of patients and controls

Parameter	IgG4-RD (*n *= 15)	SS (*n *= 16)	HC (*n *= 10)
Age (years)	62.9 ± 10.5^a^	55.9 ± 15.7^a^	40.9 ± 17.1
Sex (males/females)	6/9	2/14	5/5
Anti-SS-A antibody (%)	0^b^	93.8	n.d.
Anti-SS-B antibody (%)	0^b^	56.3	n.d.
Serum IgG4 (mg/dl)	942.2 ± 612.5	n.d	n.d.
Serum IgE (U/ml)	294.0 ± 317.8	n.d	n.d.
CH50 (U/ml)	33.6 ± 17.5^b^	50.6 ± 16.3	n.d.
IgG4/IgG in LSG (%)	62.0 ± 3.5	n.d	n.d.

Data are expressed as mean ± SD. n.d., not determined. ^a^*P *< 0.05, compared with the healthy control subjects (Mann-Whitney *U *test or Fisher Exact probability test). ^b^*P *< 0.05, compared with the SS group (Mann-Whitney *U *test or Fisher Exact probability test).

**Table 2:** Material sampling and organ involvements in patients with IgG4-RD

Case	Material sampling			Organ involvement
	**PBMC**	**CD3/CD20**	**LSG**^a^	

1	○		○	MD, lymph node, lung
2	○		○	MD, lymph node
3	○	○		MD
4	○			MD, lymph node
5			○	MD, lung
6	○	○		MD, lymph node, para-aorta swelling
7	○	○		Intraorbital mass, retroperitoneal fibrosis
8			○	MD
9			○	MD
10			○	MD
11			○	MD
12			○	MD
13			○	MD
14			○	MD, lung
15			○	MD, liver

PBMCs, peripheral blood mononuclear cells; CD3/CD20, CD3-positive T cells and CD20-positive B cells sorted from PBMCs; LSG, labial salivary gland; MD, Mikulicz disease (salivary and lacrimal glands). ^a^All LSG samples satisfied histopathologic findings of the comprehensive diagnostic criteria for IgG4-related disease (IgG4-RD) 2011.

### The cell population of CD3-positive T cells and CD20-positive B cells in PBMCs

**Figure 1:**
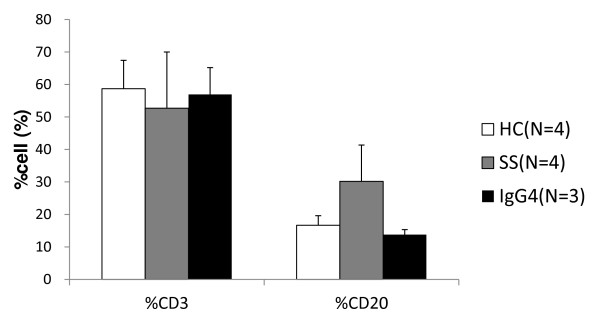
**The cell population of CD3-positive T cells and CD20-positive B cells in peripheral blood mononuclear cells**. PBMCs stained with anti-CD3 antibody and anti-CD20 antibody were analyzed and sorted with flow cytometory. HC, healthy control; SS, Sjögren syndrome; IgG4, IgG4-RD.

Figure 1 shows the cell population of CD3-positive T cells and CD20-positive B cells sorted from PBMCs of patients with IgG4-RD (*n *= 3), SS (*n *= 4), and the control (*n *= 4). The population of CD20-positive B cells in SS was higher than the others (no statistically significant difference), whereas that of CD3-positive T cells was comparable in the three groups.

### The mRNA expression levels of IgG4-specific class switch-related molecules in PBMCs, CD3-positive T cells, and CD20-positive B cells in PBMCs and LSGs

**Figure 2:**
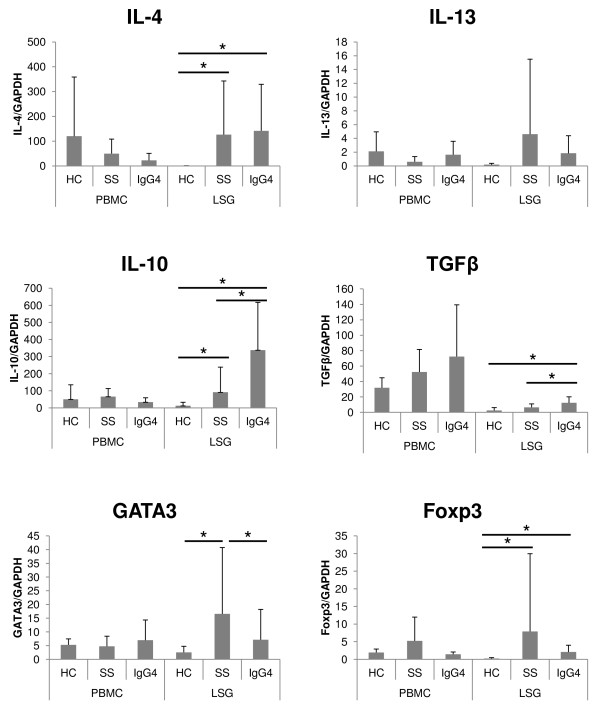
**The mRNA expression levels of IgG4-specific class switch-related molecules in PBMCs and LSGs**. The displayed mRNA expression levels are relative to the mRNA level of GAPDH, representing the internal control. Data are expressed as mean ± SD. *P < 0.05, Mann-Whitney *U *test. PBMCs, peripheral blood mononuclear cells; LSGs, labial salivary glands; HC, healthy control; SS, Sjögren syndrome; IgG4, IgG4-RD.

Figure 2 shows the mRNA expression levels of IgG4-specific class switch-related molecules in PBMCs and LSGs. The mRNA expression level of IL-4 was significantly higher in LSGs of IgG4-RD than in the control (*P *< 0.05, Mann-Whitney *U *test). Treg cytokines (IL-10 and TGF-β) were significantly higher in LSGs of IgG4-RD than SS and control (*P *< 0.05, each, Mann-Whitney *U *test). No significant differences were noted in the PBMC expression levels of various cytokines, among the three groups. In LSGs, the expression of GATA3 was significantly lower in IgG4-RD than in SS; Foxp3 was significantly higher in IgG4-RD and SS than in the control (*P *< 0.05; Mann-Whitney *U *test). However, no statistically significant difference in Foxp3 expression level was seen between IgG4-RD and SS.

No significant differences were seen in the expression levels of various IgG4-specific class switch-related molecules in CD3-positive T cells and CD20-positive B cells sorted from PBMCs, among the three groups (data not shown).

### The mRNA expression levels of IgG4-nonspecific class switch-related molecules in PBMCs, CD3-positive T cells, and CD20-positive B cells in PBMCs and LSGs

**Figure 3:**
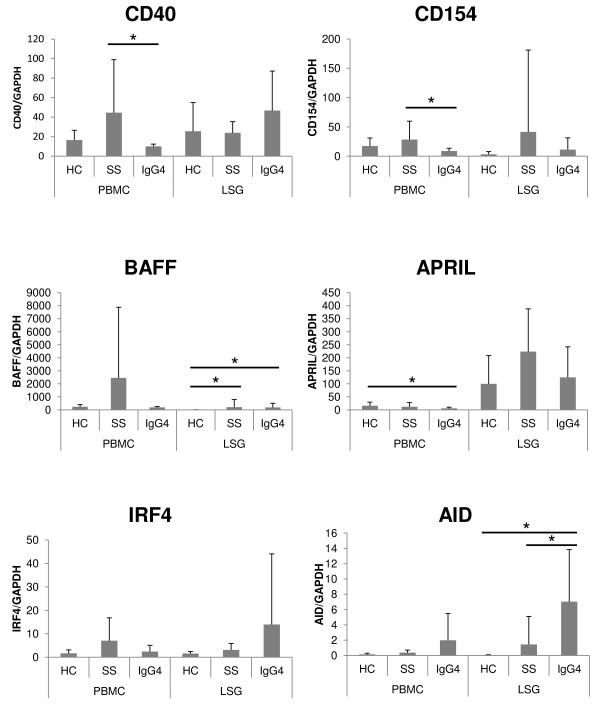
**The mRNA expression levels of IgG4-nonspecific class switch-related molecules in PBMCs and LSGs**. The displayed mRNA expression levels are relative to the mRNA level of GAPDH, representing the internal control. Data are expressed as mean ± SD. *P < 0.05, Mann-Whitney *U*-test. PBMCs, peripheral blood mononuclear cells; LSGs, labial salivary glands; HC, healthy control; SS, Sjögren syndrome; IgG4, IgG4-RD.

Figure 3 displays the mRNA expression levels of IgG4-nonspecific class switch-related molecules in PBMCs and LSGs. The mRNA expression levels of CD40 and CD154 were significantly lower in PBMCs of IgG4-RD than in SS (*P *< 0.05, each; Mann-Whitney *U *test). The expression of BAFF was significantly higher in LSGs of IgG4-RD than in the control (*P *< 0.05; Mann-Whitney *U *test). The expression of APRIL was significantly lower in PBMCs of IgG4-RD than in the control (*P *< 0.05; Mann-Whitney *U *test). The expression of AID was significantly higher in LSGs of IgG4-RD than in SS and the control (*P *< 0.05, each; Mann-Whitney *U *test).

**Figure 4:**
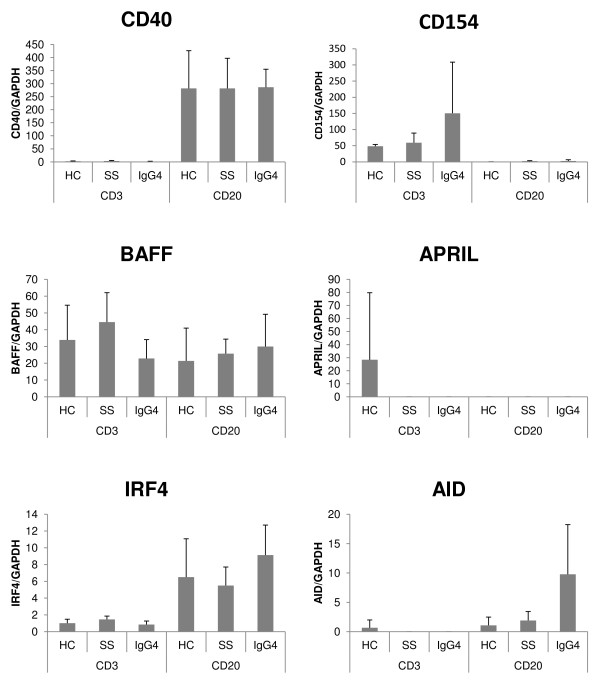
**The mRNA expression levels of IgG4-nonspecific class switch-related molecules in CD3-positive T cells and CD20-positive B cells sorted from PBMCs**. The displayed mRNA expression levels are relative to the mRNA level of GAPDH, representing the internal control. Data are expressed as mean ± SD. *P < 0.05; Mann-Whitney's *U *test. CD3, CD3-positive T cells sorted from PBMCs; CD20, CD20-positive B cells sorted from PBMCs; HC, healthy control; SS, Sjögren syndrome; IgG4, IgG4-RD.

Figure 4 shows the mRNA expression levels of IgG4-nonspecific class switch-related molecules in CD3-positive T cells and CD20-positive B cells sorted from PBMCs. In contrast to PBMCs, the expressions of CD40 in CD20-positive B cells and that of CD154 in CD3-positive T cells were comparable in the three groups. Moreover, no significant difference occurred in the expression of APRIL in CD3-positive T cells and CD20-positive B cells sorted from PBMCs, among the three groups. The expression of AID in CD20-positive B cells from IgG4-RD was higher than others (no statistically significant difference).

## Discussion

The clinical and pathologic features of patients with IgG4-RD participating in this study (such as low frequencies of anti SS-A antibodies and anti SS-B antibodies, high serum IgG4 levels, high IgG4/IgG in LSGs, and low CH50 levels) accord with previous reports [1].

We revealed the mRNA expression levels of IgG4-specific and nonspecific class switch-related molecules in both PBMCs and LSGs of IgG4-RD, and then, these levels were compared with those measured in patients with SS and controls. We focused on the molecules with different expression levels in IgG4-RD than in SS and control, with the assumption that these molecules could be IgG4-RD-specific pathogenic factors.

Among IgG4-specific class switch-related molecules, the expression levels of Treg cytokines (IL-10 and TGF-β) in LSGs of IgG4-RD were significantly higher than in SS and the control, in agreement with previous reports [4,5]. We

assume that these cytokines might be produced by Treg cells in LSGs of IgG4-RD. According to this speculation, the mRNA expression level of Foxp3, which is a master transcriptional factor for Treg cells, was higher in LSGs of IgG4-RD than in the control. We also showed that the expression of GATA3 was significantly lower in LSGs of IgG4-RD than in SS. It is reported that in salivary glands of SS, Th2 cells were detected as well as Th1 cells, and could contribute to activation of B cells through production of IL-4 [17]. Therefore, the lower mRNA expression of GATA3, a master transcriptional factor for Th2 cells, in IgG4-RD than in SS might be upregulation in SS but not downregulation in IgG4-RD. In SS, impaired Treg response or imbalance between a Treg response and a proinflammatory response might cause upregulation of Th1 and Th2 response that contributed to the pathophysiology of SS. Conversely, in IgG4-RD, upregulation of the Treg response itself could contribute to pathogenesis. Interestingly, it was previously reported that IL-10 enhanced IgG4 production from IL-4-stimulated PBMCs *in vitro *[9]. Therefore, in LSGs of IgG4-RD, IL-10 might induce IgG4-specific class-switch recombination, and TGF-β might cause tissue fibrosis [5,11]. Thus, Treg cytokines (IL-10 and TGF-β) might play important roles in IgG4-specific class-switch recombination and fibrosis, which are characteristic features of IgG4-RD.

Among IgG4-nonspecific class switch-related molecules, the expression of AID was significantly higher in LSGs of IgG4-RD than in SS and the control. The roles of IgG4-nonspecific class switch-related molecules such as AID in the pathogenesis of IgG4-RD have not been reported previously. The present study showed that the expression level of AID was different in IgG4-RD than in SS and the control. AID is essential for nonspecific immunoglobulin class-switch recombination (from IgM to IgG1, IgG2, IgG3, IgG4, IgA, and IgE) [12-14]; thus, upregulation of AID could also contribute to upregulation of IgG4-specific class-switch recombination along with IL-10 in LSGs of IgG4-RD.

We showed that in SS, the population of CD20-positive B cells in PBMCs was much more than IgG4-RD and the control (statistically not significant). Therefore, CD40 and CD154 mRNAs in PBMCs of SS were highly expressed compared with that in IgG4-RD. These findings might be due to systemic B-cell activation in SS patients instead of local B-cell activation in IgG4-RD. Moreover, in CD3-positive T cells and CD20-positive B cells sorted from PBMCs, we found no IgG4-specific, -nonspecific, class switch-related molecules with different expression levels in IgG4-RD than in SS and controls.

These observations on the expression levels of AID and Treg cytokines suggest that IgG4 class-switch recombination in IgG4-RD was upregulated mainly in the tissues of the affected organs. However, the mechanism of upregulation of Treg cytokines in IgG4-RD is unknown. Treg response itself primarily regulates the immune response and inflammation [17]; therefore, increased Treg cytokines could be a nonspecific response to dampen the inflammation, which could increase the IgG4 class switch in the tissues.

## Conclusion

This study showed different expression levels of IgG4 class switch-related molecules in LSGs than in PBMCs of IgG4-RD. In LSGs of IgG4-RD, increased Treg cytokines (IL-10 and TGF-β) might play pathogenic roles in IgG4-specific class-switch recombination and fibrosis. AID was also increased in LSGs of IgG4-RD and could contribute to upregulation of IgG4-specific class-switch recombination along with IL-10 in LSGs. Thus, overexpression of IL-10, TGF-β, and AID in LSGs might play important pathogenic roles in IgG4-RD. IgG4 class-switch recombination seemed to be mainly upregulated in affected organs.

## Abbreviations

AID: activation-induced cytidine deaminase; APRIL: a proliferation-inducing ligand; BAFF: B cell-activating factor belonging to the tumor necrosis factor family; IgG4-RD: IgG4-related disease; IRF4: interferon regulatory factor 4; LSG: labial salivary gland; PBMC: peripheral blood mononuclear cell; SS: Sjögren syndrome.

## Competing interests

The authors declare that they have no competing interests.

## Authors' contributions

All authors have read and approved the manuscript for publication. Each author took part in the design of the study, contributed to data collections, participated in writing the manuscript, and all agree to accept equal responsibility for accuracy of the contents of this article.
